# Frontline healthcare worker barriers impacting tuberculosis service demand and access in high burden settings: a scoping review

**DOI:** 10.7189/jogh.16.04243

**Published:** 2026-09-18

**Authors:** Samyra R Cox, Colleen F Hanrahan, Aye Hnin Moe, Kate Shearer, Canice Christian, Sejal Sethi, Mollie Hudson, Manami Ueshima, Kalee Singh, Jane Dawson, Ketholelie Angami, Nyiko Kubai, Mangala Namasivayam, Priya Shete, Jonathan E Golub, Andrew D Kerkhoff

**Affiliations:** 1 Johns Hopkins Bloomberg School of Public Health, Baltimore, USA; 2 Center for Tuberculosis Research, Johns Hopkins University, Baltimore, USA; 3 Center for Tuberculosis, University of California San Francisco, San Francisco, USA; 4 Access to Rights and Knowledge Foundation, Kohima, Nagaland, India; 5 Afrocab Treatment Access Partnership, Lusaka, Zambia; 6 APCASO, Bangkok, Thailand; 7 Department of Epidemiology and Biostatistics, University of California, San Francisco, USA; 8 Division of HIV, ID, Global Medicine, San Francisco General Hospital, University of California San Francisco, San Francisco, USA

**Keywords:** tuberculosis, health personnel, health services accessibility, health services research, review

## Abstract

**Background:**

Extensive literature exists on health-seeking behaviour and barriers related to the uptake of tuberculosis (TB)-related services in high TB burden settings. However, prior reviews have focused on patient- and health facility/systems-level barriers to TB-related care. In this scoping review, we aimed to synthesise frontline healthcare worker (FLHCW)-related barriers that limit individuals’ demand for, access to, and retention in TB services.

**Methods:**

We searched PubMed for studies describing FLHCW-related barriers that impact demand for and/or access to TB services (preventive treatment, active case finding, diagnosis and treatment initiation, treatment completion, and post-TB care). We included studies that were published between 2010 and 2025 in English, and that reported on original research findings from the World Health Organization lists of high TB burden countries. Two reviewers independently conducted title and abstract screening, and full-text review. We narratively summarised barriers by theme and applied the Capability, Opportunity, Motivation-Behavior (COM-B) framework to facilitate interpretation.

**Results:**

We screened a total of 5,306 records, and included 189 studies, most of which were from India, South Africa, and Ethiopia. Among the included studies, most reported barriers were for doctors (n = 101, 53%) and nurses (n = 82, 43%). Studies identified FLHCW-related barriers at every step of the care cascade except for post-TB care. Knowledge and skill gaps leading to missed opportunities to engage or retain individuals in care were the most frequent barriers identified (n = 128 studies). Other major themes included provider-patient communication, discrimination and stigma from FLHCWs, and motivation to provide quality TB clinical care. Mapping barriers onto COM-B identified actionable targets for strategies to improve TB care.

**Conclusions:**

FLHCW-related barriers to TB care are pervasive and multifaceted, affecting the care cascade from prevention to diagnosis to treatment initiation and completion. Addressing these barriers will require multi-component implementation strategies that are context-specific and designed to empower FLHCWs as drivers of care.

Tuberculosis (TB) is a preventable and curable disease but remains the leading infectious cause of death worldwide. In 2024, an estimated 10.7 million people developed active TB disease and 1.23 million people died from the disease [1]. Reducing the burden of disease with the tools currently available depends on both proactive health-seeking behaviour by affected community members and the delivery of high-quality services by providers supported by strong health systems. Extensive literature has examined health-seeking behaviour and barriers related to the uptake of TB services [2–28]. While existing reviews on the topic cover a range of TB services, they primarily focus on patient-level and health facility/system-level barriers to TB care. However, frontline healthcare workers (FLHCW) play a critical role in determining individuals’ demand for, access to, and retention in timely and high-quality TB services.

FLHCW knowledge gaps, attitudes, and competing priorities can create barriers that discourage care-seeking behaviour [29]. Understanding these provider-side barriers is crucial for developing effective strategies to increase engagement with TB services, which may lead to greater uptake of TB preventive treatment, improved TB diagnostic and treatment outcomes, and long-term reductions in TB incidence, morbidity, and mortality. Despite growing recognition of the need to improve health service delivery by measuring and addressing client concerns [30], the barriers related to FLHCWs are often underexamined through a structured lens.

To address these gaps, we synthesised the literature on FLHCW-related barriers that may impact individuals’ engagement in TB services across the care cascade in high TB burden countries. By identifying and organising provider-side barriers through a behaviour change framework, we aimed to inform the design of effective implementation strategies for both routine TB services and innovations.

## METHODS

We conducted this scoping review as part of the Developing a Global Framework to Enhance Demand, Access, and Seeking of TB-related Healthcare Services (TB-DASH) project. We followed the Joanna Briggs Institute guidance for scoping reviews [31], and our findings are reported in accordance with the PRISMA-ScR [32]. Our screening, data extraction, and synthesis procedures were pre-specified in a study protocol (Appendix S1 in the **Online Supplementary Document**).

### Inclusion and exclusion criteria

We included studies if they described FLHCW-related barriers that impact demand for and/or access to TB services, were published between 2010 and 2025 in English, and reported on original research findings from one or more high TB burden countries. This time period was chosen because major diagnostic advancements, such as Xpert Mycobacterium tuberculosis/Rifampicin, were introduced in 2010, which substantially changed TB service provision [33].

We defined FLHCW as cadres of health workers who provide direct medical care, including physicians, nurses, clinical officers and associates, pharmacists, and community health workers. In this review, ‘impacting demand and/or access’ refers to FLHCW-related actions or inactions that delay care, discourage care-seeking, reduce service quality, or contribute to incomplete or interrupted TB care (*i.e.* hinder engagement and/or retention). Furthermore, we considered TB services across the entire care cascade: TB preventive treatment, active case finding, diagnosis and treatment initiation, treatment completion, and post-TB care. Studies that did not specify a care cascade step were categorised into ‘general TB care’.

We defined ‘high TB burden country’ based on the World Health Organization’s (WHO) lists (49 unique countries across the high TB, TB/HIV, and drug-resistant TB lists) [34]. High TB burden countries often face dual challenges of limited resources and high caseloads (systems-level barriers), making the role of FLHCWs even more critical in closing access and equity gaps. Focusing on these settings provides insights into the contexts where health worker-related barriers may be most pronounced, and where targeted strategies may yield the greatest impact.

We excluded studies that only reported on patient-level or health systems-level barriers, those that did not present original research (*e.g.* protocols, systematic reviews, editorials, and/or commentaries), those that were abstracts or conference proceedings only, or were not available in English [33].

### Selection of studies

We searched PubMed up to 24 March 2025, using search terms covering the concepts of TB and high TB burden countries, barriers, and healthcare workers. A complete list of search terms was developed in consultation with a Johns Hopkins University information specialist and further refined through expert consensus, aligning with other work streams within the TB-DASH project (Appendix S1 in the **Online Supplementary Document**). We uploaded the resulting titles and abstracts into Covidence (Veritas Health Innovation, Melbourne, Australia), and removed duplicates. To ensure consistency in interpretation, screening was done in pairs, with seven reviewers (KS, CH, AHM, CC, SS, MH, SC) participating at the title and abstract review, and five reviewers (KS, CH, AHM, SS, SC) in the full-text review. To better distinguish FLHCW-level from health system-level barriers, we established operational definitions at the full-text review stage to improve consistency in how reviewers excluded articles that only addressed health system-level barriers. Specifically, we categorised environmental and structural factors (*e.g.* staff shortages, supply/funding shortages, referral coordination issues) as health system-level barriers. Discrepancies between two reviewers were reconciled by a third reviewer (SC) using the same definition.

### Data abstraction

After conducting the full-text review, reviewers discussed emerging themes from the literature, which we then incorporated into our data abstraction tool to facilitate the categorisation of barriers. We finalised the data abstraction tool with input from three community technical advisors (KA, NK, MN) working in the TB sector in high-burden countries. We integrated the data abstraction tool into Covidence and included title, author, year of publication, location, study design, TB service(s), drug resistance, FLHCW cadre, care-seeking population, and FLHCW-related barriers. The first five studies were reviewed by two reviewers (KS, CH) for consistency, and the data were abstracted independently thereafter. Considering the interpretive nature of barrier categorisation, as an additional quality check, abstracted data were then reviewed by one of the more experienced reviewers (KS, CH, AHM). Discrepancies in interpretation were discussed and then revised for consistency. A few authors (CC, CFH, JEG) on this review were also authors on included studies – these authors were not involved in the data abstraction of their own studies. Quotations from qualitative studies were selected illustratively to exemplify identified themes, with source study design, setting, and population described alongside each quotation.

### Data synthesis

We conducted a narrative synthesis of the extracted qualitative and, due to heterogeneity in the reported endpoints, quantitative data collected from both individuals seeking care and FLHCWs. We treated findings from both study designs as equivalent contributions to the descriptive synthesis, as we aimed to map the breadth of reported barriers rather than to assess effect sizes or causal relationships.

Our primary analysis entailed summarising barriers by theme across the TB care cascade steps and selecting quotes from qualitative studies to represent the perspectives of both individuals seeking care and FLHCWs. Thematic categories were mutually exclusive, although some overlap is inherent (Appendix S2 in the **Online Supplementary Document**). We also used the Capability, Opportunity, Motivation Behavior (COM-B) framework for behaviour change to systematically categorise types of FLHCW-related barriers and to support the development of targeted, theory-informed implementation strategies. COM-B is a synthesis of 19 behaviour change frameworks and has been applied widely across different health domains to inform individual-level behaviour change strategies and improve public health outcomes [35]. Following completion of thematic synthesis, two reviewers independently mapped themes onto COM-B domains, guided by domain definitions from the original COM-B publication [35], with discrepancies resolved through discussion.

### Patient and public involvement statement

The authors have a mixture of lived experiences as both FLHCW and individuals seeking or receiving care in high TB burden settings; however, a significant proportion live and work in the USA (a low TB burden setting). For this reason, we have augmented our experiences through consultation with three community technical advisors (KA, NK, MN). The community technical advisors involved in this project are members of the public who are actively engaged in service provision and policy for TB in a high TB burden country. These three advisors were involved in the methods and interpretation of the results.

## RESULTS

**Figure 1:**
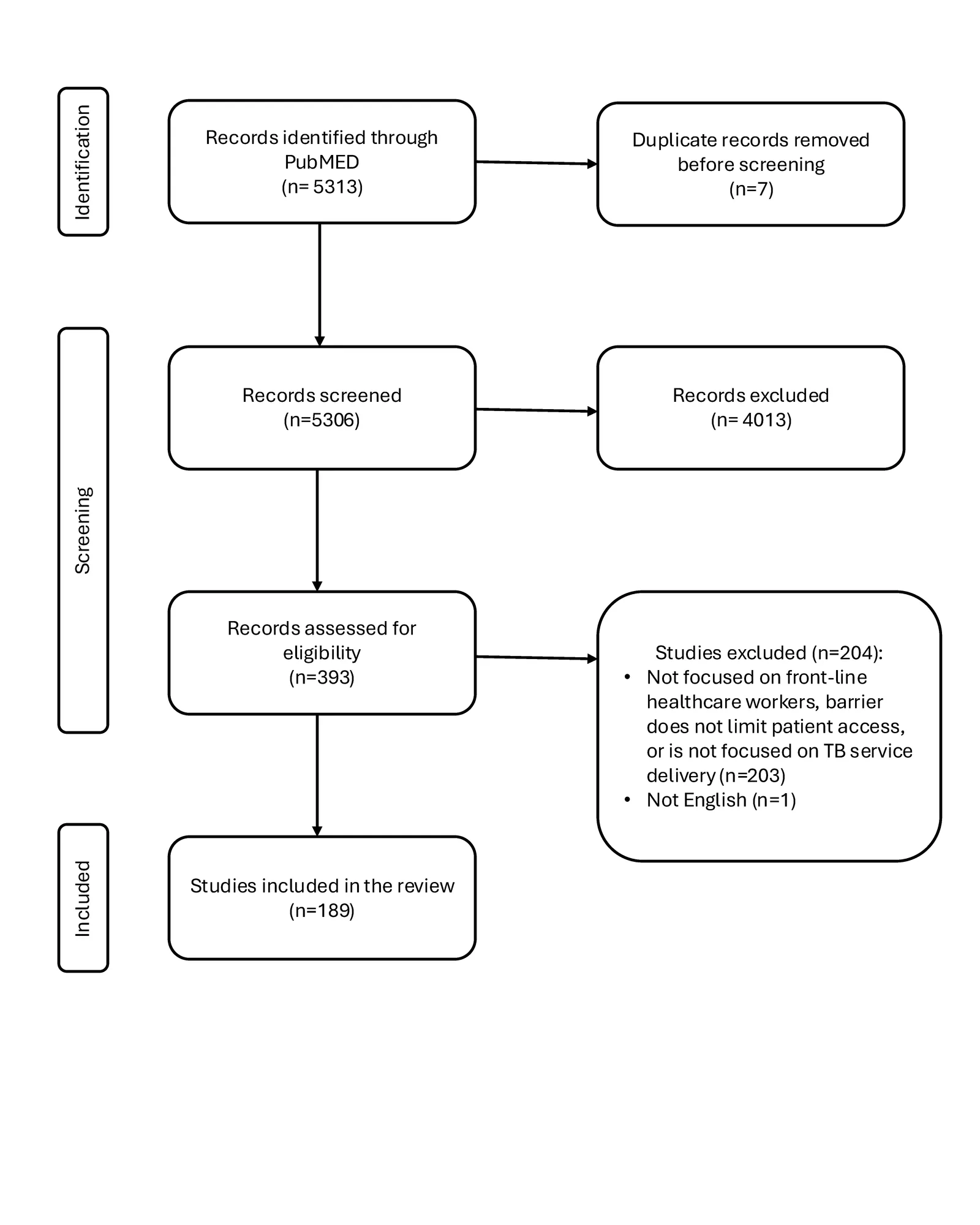
PRISMA flow diagram for the included studies.

We screened a total of 5,306 studies and selected 393 for full-text review, of which 189 met the inclusion criteria (**Figure 1**; Appendix S4 in the **Online Supplementary Document**). Included studies covered nearly the entire spectrum of the TB care cascade, reporting on relevant barriers related to TB preventive treatment (n = 33, 17%), active case finding (n = 19, 8%), diagnosis and treatment initiation (n = 69, 37%), and treatment completion (n = 57, 30%). However, no studies reported relevant barriers during the provision of post-TB care. Moreover, 43 studies (23%) discussed TB care generally, without specifying an individual care cascade step; 37 studies (20%) identified barriers that were specifically related to drug-sensitive TB services; 45 studies (24%) focused on drug-resistant TB services; 62 (33%) studies described risk factors for individuals seeking care and community members, including living with HIV (n = 37, 20%), household contact exposure (n = 13, 7%), diabetes (n = 2, 1%), malnutrition (n = 1, 0.5%), mining exposure (n = 1, 0.5%), migration (n = 1, 0.5%), and pregnancy (n = 4, 2%). Qualitative methods (*e.g.* semi-structured in-depth interviews, focus group discussions, ethnography) were used in most studies (n = 155, 82%), while 55 (29%) studies used quantitative methods. We categorised barriers as related to knowledge and skills; communication and attitudes; discrimination and stigma; or motivation to provide quality care (**Table 1**). Knowledge and skills gaps were the most commonly reported barriers (n = 128 studies, 68%), which were consistently reported across the TB care cascade. Challenges with healthcare worker communication and attitude, rapport, and/or trust were the next most frequently reported (n = 81 studies, 43%) and also spanned the care cascade. Discrimination and stigma from healthcare workers, often manifesting as fear and avoidance of persons with TB (n = 33 studies, 17%), were present at the diagnosis and treatment steps. FLHCW motivation to provide quality TB clinical care was identified as a barrier to individuals accessing care at each step but was less frequently reported (n = 29 studies, 15%).

**Table 1:** Summary of FLHCW-related barriers reported for each step of the TB care cascade among 189 included publications

	TB preventive treatment	Active case finding	Diagnosis and treatment initiation	Treatment completion	General TB care
**Knowledge and skills**	Lack of knowledge on TPT prescribing; ambiguity/changes in guidelines leading to uncertainty over TPT eligibility; drug-resistance concerns; scepticism about TPT efficacy; missed opportunities for prescribing or refilling TPT; difficulties ruling out active TB disease before prescribing TPT	Knowledge deficits related to risk groups who should receive systematic screening; inadequate skills related to screening, presumptive case identification, or contact investigation; missed diagnosis opportunities due to low clinical suspicion among risk groups	Knowledge deficits related to TB symptoms, sample collection, appropriate diagnostic tests, diagnostic algorithms, and diagnostic test interpretation; lack of knowledge of standard treatment regimens; difficulties identifying people with presumptive TB who require diagnostic testing; treating with broad-spectrum antibiotics without assessing TB symptoms	Knowledge deficits related to TB management and DOTS; inadequate familiarity with TB treatment guidelines; poor knowledge related to treatment duration, action for non-adherence and adverse event management; inadequate skills related to patient support techniques	Knowledge deficits related to stigma-reduction, DR-TB care, DOTS, and integrated HIV/TB care; inadequate familiarity with TB guidelines; poor knowledge about how to implement community-level TB interventions (health promotion, contact tracing, treatment support); inadequate knowledge related to procedures for private sector engagement in referrals for TB
Number of studies	30	15	61	15	23
**Communication and attitudes**	Poor communication about the need for TPT and duration of treatment; negative staff attitudes reducing demand for TPT	Poor communication when screening for symptoms and referring for further diagnostic workup; perception that data collection is more important than rapport building	Negative, rude, uncaring or hostile attitudes reducing demand for diagnosis or treatment; unclear, vague or limited communication about diagnostic testing, treatment procedures and side-effects; cultural/linguistic barriers in engaging with individuals in care	Lack of interaction and responsiveness when the individual in care faces TB treatment issues; negative attitudes discouraging care engagement; inadequate counselling on TB disease severity, duration of treatment, adherence, and adverse event management	Poor communication about TB disease and its implications; inability to speak preferred language of those in care; inadequate FLHCW counselling about hospital discharge, referrals and treatment adherence
Number of studies	6	5	17	41	17
**Discrimination and stigma**	NA	NA	Discrimination during diagnosis/treatment initiation procedures based on culture, ethnicity or language; fear of transmission from individuals with presumptive or confirmed TB; stigma toward people diagnosed with TB and living with HIV or diabetes; mistreatment or abuse of individuals seeking care	Discrimination against individuals with DR-TB in particular; stigmatising behaviour and practices (*e.g.* refusing to touch an individual); use of stigmatising language directed at pregnant women with DR-TB	Fear-based discrimination against individuals with DR-TB; use of stigmatising language (*e.g.* calling those lost to care ‘defaulters’); mistreatment or abuse of those seeking care; staff avoidance of DR-TB wards and individuals with DR-TB
Number of studies	0	0	6	11	17
**Motivation to provide quality care**	Lack of prioritisation related to TPT provision; concern about stock-outs leading to a reluctance to prescribe TPT	Inadequate incentives to proactively screen for TB among high-risk populations, including household contacts; competing priorities, especially when yield of active case finding for TB is perceived as being low	High workload, long hours, lack of opportunities leading to low motivation to deliver quality care; lack of willingness to refer for further evaluation; perception that those in care lack willingness to follow care recommendations	Disinterested and negligent in support and monitoring of those in care; lack of incentive to provide treatment support services	Lack of motivation to gain skills due to limited promotion opportunities; unwillingness and fear towards DR-TB care provision; lack of orientation on assigned roles and responsibilities; frequent updates to guidelines reduce enthusiasm to learn of changes
Number of studies	5	8	6	3	8

DOTS – directly observed therapy short-course, DR-TB – drug-resistant TB care, FLHCW – frontline healthcare worker, HIV – human immunodeficiency virus, NA – not applicable, TB – tuberculosis, TPT – TB preventive treatment

*One paper may describe multiple barriers.

### Knowledge and skills

A lack of or incorrect knowledge, skills, and beliefs among providers was noted by individuals seeking care, their family members, and providers themselves. This important gap led to missed opportunities to engage or retain individuals in TB care and was the most commonly identified FLHCW-related barrier to healthcare-seeking behaviour. This barrier was reported across all FLHCW cadres, including doctors, nurses, pharmacists, TB focal point persons, and community health workers, and at all stages of the TB care cascade. Individuals in care in particular noted knowledge gaps affecting timely diagnosis:

Look, the worst part was the delay for the right diagnosis. I think that it could be improved. Like, an immediate diagnosis, correct diagnosis. I think I wouldn’t have to spend so much time... I think I would’ve been at the end of treatment. I’m practically crawling again. *– individual with MDR-TB in Brazil (unspecified sex) [**36**]*

Among FLHCWs, knowledge and skills gaps were attributed to insufficient education, a lack of ongoing or updated training, or unclear or incomplete care guidelines. These gaps contributed to missed or delayed diagnoses or a perceived lower quality of care, resulting in losses to follow-up.

*Patients are repeatedly treated with antibiotics, which usually leads them to complain because of exacerbation of their symptoms. This will encourage patients to make self-referral decisions to visit other costly healthcare facilities. A few days after visiting those health facilities, they come back with a positive TB result. This implies that we are missing infectious TB cases due to knowledge/skill gaps.* This usually happens among [FLHCW] who have graduated from private colleges. Many people say that [health workers] who graduated from private colleges did not get adequate supportive supervision and had fewer clinical practice hours before graduation. *– FLHCW in Ethiopia (male) [**37**]*

Previously we were not sure… now there are [TB preventive treatment durations of] three years… one year. We were all not sure for how long… We thought rather than making mistakes, let’s not give it out. But now [that we’ve completed the workshops] there is a change. *– FLHCW in South Africa (unspecified sex) [**38**]*

### Communication and attitudes

We identified poor communication skills, a lack of rapport between individuals in care and FLHCW, and/or a negative attitude on the part of FLHCW as barriers to care. This barrier was reported among a wide range of cadres and at all stages of the TB care cascade. Individuals in care particularly cited negative attitudes by providers as reasons to either delay or avoid seeking care, or to disengage from care.

I would like to continue with my treatment, I just don’t want to be shouted at because that is also working me on my nerves. I wish they can talk to this nurse because she is making me angry sometimes.” – individual with TB in South Africa (unspecified sex) *[**39**]*

The staff is just rough… The last time I was there, I wasn’t feeling well. They said something I just couldn’t understand…they said, ‘you’re the problem. After doing your things out there, you come to us coughing and almost dying.’ Imagine I am in pain and then someone speaks like that to me. *– community member in Zimbabwe (male) [**40**]*

### Discrimination and stigma

We identified discrimination and stigma on the part of the FLHCW as a reason for avoiding or disengaging from care. Many people recalled being blamed for their illness, made to feel dirty, or accused of spreading TB illness among FLHCW. Some studies noted discrimination due to race, ethnicity, residence, or other socio-demographic characteristics. Some FLHCW noted discrimination and stigma present among colleagues and other cadres of workers. No studies reported on discrimination or stigma within TB preventive treatment or active case finding activities; however, multiple studies identified this barrier at later stages of the TB care cascade.

I was having problems because of medicines. When I went to see the doctor, my face was already covered with the burqa (Muslim women cover their face), but they asked- ‘why did you not cover your mouth? You have MDR-TB.’ I said my face is covered. Then they said lot of things. Even the sister (nurse) said a lot [unkind words] while giving injections. Though I tried to speak nicely, they were not speaking nicely. *– individual with TB in India (female) [**41**]*

Some healthcare providers do not know how to talk to patients in a polite and respectable manner. They sometimes shout at clients who come to the facility for no reason. They think we shall contaminate them with the disease (TB). They don’t even want to draw close to us when talking to us and we must get drugs through that small window. *– individual with TB in Zambia (unspecified sex) [**42**]*

### Motivation to provide quality care

FLHCW identified low motivation to provide quality TB care among providers as an ambivalence or profound lack of attention towards their job responsibilities. Causes of low motivation were varied, including insufficient incentives, lack of training, an ever-changing landscape of clinical guidelines, or burnout due to high patient volume or long working hours. Low motivation was distinctly tied to a decrease in the quality of clinical care, leading to missed opportunities at all steps of the care cascade to engage or retain individuals.

From the provider’s side is a lack of motivation. Imagine you are going to attend to a patient every day for … 20 months, that means you abandon almost all you have to do for that patient. And in a situation where salaries are not forthcoming…Most of the providers in the face of no salaries will … envy what the patients are getting… So, from the provider side this is one of the major challenges. *– FLHCW in Nigeria (unspecified sex) [**43**]*

### Synthesis using COM-B

**Figure 2:**
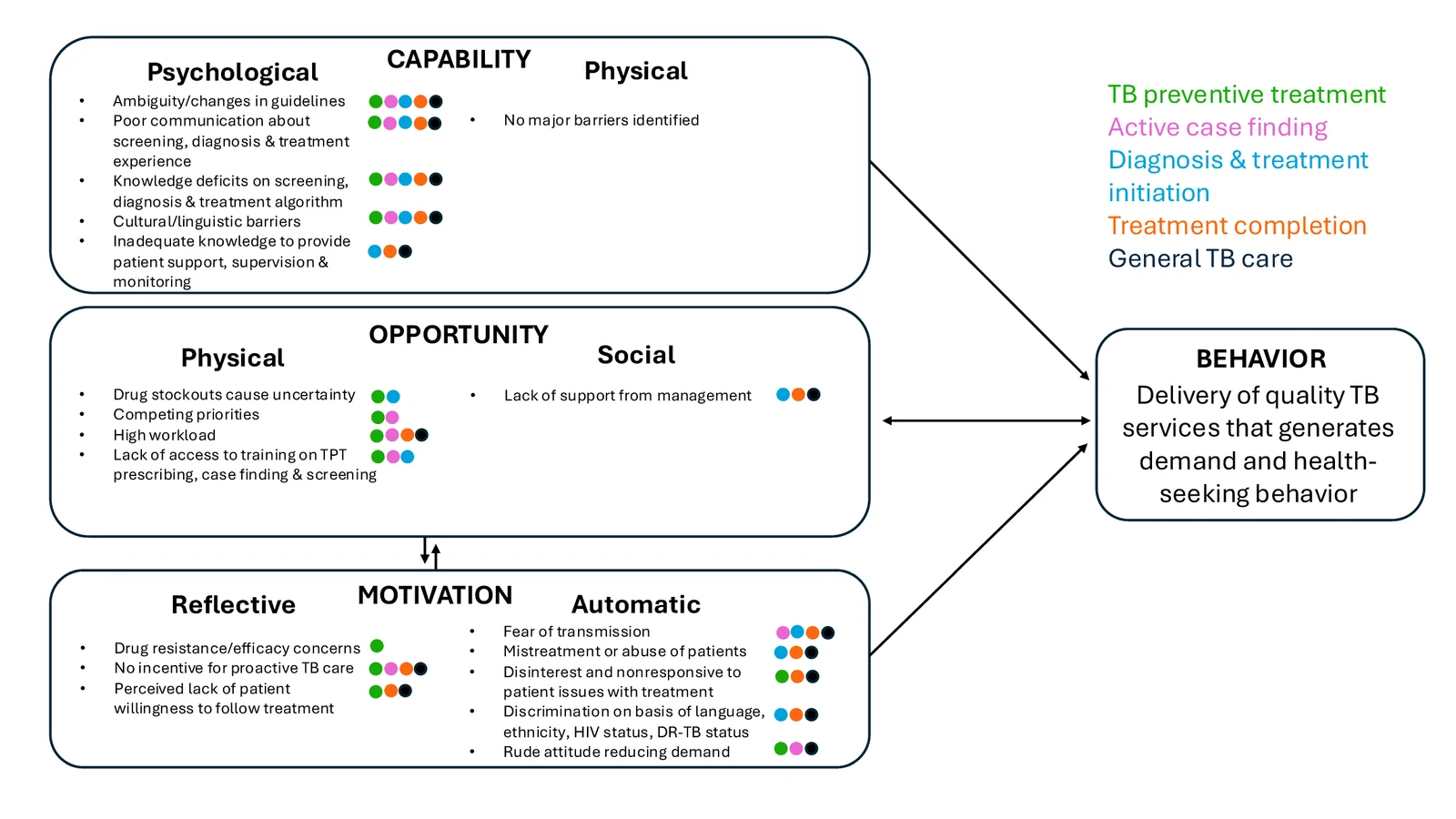
Applying the COM-B Framework to understand FLHCW-related barriers to TB care in high TB burden countries. Green indicates TB preventive treatment, purple shows active case finding, blue indicates diagnosis and treatment initiation, orange shows treatment completion, and black indicates general TB care. FLHCW – frontline healthcare worker, TB – tuberculosis.

Using the COM-B framework, we identified actionable targets for strategies to improve TB care (**Figure 2**). Our analysis revealed that psychological capability-related barriers were most prevalent across all cascade steps, including knowledge gaps in TB preventive treatment eligibility criteria, diagnostic algorithms, and treatment regimens, alongside widespread training deficiencies and poor provider communication skills. Physical and social opportunity barriers manifested as resource constraints (*e.g.* uncertainty caused by drug stock-outs, high workload, competing priorities) and lack of support from management. Motivation barriers encompassed both reflective processes (*e.g.* scepticism about TB preventive treatment efficacy, perceived lack of willingness of those in care to follow treatment recommendations, inadequate incentives) and automatic responses (*e.g.* negative provider attitudes, fear of TB transmission, pervasive stigma and discrimination, with individuals with drug-resistant TB experiencing the most severe stigmatisation through avoidance behaviours, derogatory language, and refusal of physical contact by FLHCWs), with these fear-based reactions being most pronounced during treatment phase. The distribution of barriers varied across the TB care cascade, with TB preventive treatment provision being primarily limited by capability and reflective motivation barriers, active case finding by opportunity-related barriers, and diagnosis and treatment initiation facing multidimensional challenges spanning five of the six COM-B sub-components.

## DISCUSSION

Prior to our study, the literature on FLHCW-related barriers to people demanding and accessing TB services had not been synthesised using a structured approach. Several themes were consistent across steps of the TB care cascade, including the need for deeper knowledge and additional skills related to TB prevention, diagnosis, and treatment initiation, adherence, and treatment completion. In addition, poor provider–patient communication and attitudes, a lack of rapport and trust between the FLHCW and those in care, and FLHCW stigma or discrimination towards people with presumptive or confirmed TB discouraged people from seeking TB care or from remaining engaged in care.

Our synthesis of over 180 qualitative and quantitative studies from TB-affected communities could help inform the design and testing of targeted implementation strategies that address provider-side barriers. We have included both patient and FLHCW perspectives, making our review relevant for the development of strategies aimed at eliciting behaviour change among FLHCSs, or programmes which the FLHCWs implement themselves among patients and communities. Our results suggest that these strategies should be multifaceted and not limited to addressing only one category of barriers identified here. For example, FLHCWs could be the target of enhanced training programmes to address inadequate knowledge and skills, which should be designed with input from the FLHCWs to ensure relevance and engagement, and should include modules beyond clinical guidelines, such as effective communication with those in care based on local social and cultural norms. While we identified clear themes across diverse settings, qualitative data highlighted that the components of implementation strategies that address FLHCW-related barriers may be more effective if they are context-specific and incorporate patient perspectives.

FLHCW barriers to quality TB care mirror those identified in similar settings within other healthcare service domains. For example, in a systematic review of barriers to maternal care in the African setting, a lack of experienced and knowledgeable healthcare professionals and intimidating attitudes from FLHCW were identified as important barriers to care [44]. Similar findings have been seen for HIV, malaria and non-communicable diseases [45–47]. The striking consistency of FLHCW-related barriers across diverse health service domains – from TB and HIV care to maternal health services and non-communicable disease management – underscores that many of these are not TB-specific challenges. Rather, most of the barriers we summarised reflect failures in health workforce development, necessitating comprehensive health system reforms driven by sustained political commitment. System-level reform is needed to address the fundamental issues of inadequate skills, poor supervision, insufficient resources, and weak organisational support that undermine quality care delivery across disease areas in low-income settings [30].

The breadth and complexity of identified barriers highlight the need for multi-level, theory-informed implementation strategies that move beyond traditional training-focused approaches to improving TB care. Our COM-B analysis of the literature suggests that training alone, while necessary, is insufficient. Despite widespread training requirements for FLHCWs, the persistence of knowledge and skill gaps suggests that current approaches are inadequate. More effective training programmes utilising adult learning principles, skills-based practice, and ongoing mentorship may help address individual capability barriers [48–50]. However, the prevalence of opportunity and motivation barriers demands complementary systems-level strategies, including workflow redesign to reduce workload burden, supply chain strengthening to ensure drug availability, and organisational culture change initiatives to address stigma and discrimination [51].

Future research should prioritise hybrid effectiveness-implementation trials that simultaneously evaluate clinical outcomes and implementation strategies [52], with particular attention to developing and testing strategies that address automatic motivation barriers (*e.g.* fear, mistrust, stigma) and social opportunity barriers (*e.g.* lack of supervisory support and mentorship), which have received less attention in TB implementation research despite their substantial impact on care quality. Given the differential barrier profiles across cascade steps, adaptable implementation strategies that could be tailored to local contexts while maintaining core behaviour change components appear essential. Most importantly, the contextual reality of providing high-quality TB care in high-burden, low- or middle-income settings requires solutions that are practical, affordable, and sustainable – with potential to have cross-cutting impact beyond TB care alone.

### Limitations

This systematic review has several limitations. First, differentiating between health systems-level barriers and FLHCW-level barriers is challenging, as many FLHCW barriers stem directly from systems-level constraints. For example, missed opportunities for TB diagnosis, where healthcare workers fail to recognise TB symptoms or request a TB test, may be due to understaffing, limiting FLHCW capacity to conduct thorough TB screening. While this distinction is somewhat artificial, we established category definitions during the review stage to ensure consistency. Further, the ‘physical opportunity’ domain of COM-B is specifically designed to capture environmental and structural factors that constrain behaviour, and allowed us to acknowledge and systematically organise health systems-level barriers. Second, studies solely reporting on health systems-level barriers, such as lack of access to TB diagnostics, medication stock-outs, and healthcare worker shortages, were excluded. We recognise these represent major challenges in many high-burden settings and that FLHCWs require robust and reliable infrastructure and strong systems-level support to provide quality care. Third, we limited our search to the specific countries named in the WHO’s high-TB burden lists for TB, HIV/TB, and drug-resistant TB to improve relevance to settings where solutions are most urgently needed. Thus, studies conducted in other low- and middle-income countries would have been missed by our search terms, and the distribution of identified barriers may reflect the concentration of research studies by country rather than true geographic prevalence. Fourth, we limited our review to PubMed for pragmatic reasons, which may have resulted in missed studies, particularly those disseminated outside traditional biomedical journals in high TB burden settings. Restriction to English-language publications may have introduced language bias, potentially excluding relevant evidence from high TB burden countries where research is more frequently published in other languages. Despite these limitations, we believe that thematic saturation of FLHCW barriers limiting care engagement and retention was achieved. However, more than 50% of the evidence originated from three countries (India, South Africa, and Ethiopia), highlighting the need for greater diversity of evidence from across high-burden settings. Finally, we did not identify any relevant studies on post-TB care, a relatively new area of research within the TB field.

## CONCLUSIONS

We highlight important FLHCW-related barriers hindering patient and community member demand for, uptake of, and retention in quality TB care across high TB burden settings globally. These findings could inform the design of targeted, theory-driven multi-component implementation strategies that address these barriers and ultimately improve TB prevention, diagnosis, and treatment outcomes for affected individuals and communities.

## Additional material


Online Supplementary Document

## Data Availability

**Data availability:** The study data are available from the corresponding author upon reasonable request.

## References

[1] World Health Organization. Global tuberculosis report 2025. Geneva, Switzerland: World Health Organization; 2025. Available: https://www.who.int/teams/global-programme-on-tuberculosis-and-lung-health/tb-reports/global-tuberculosis-report-2025. Accessed: 18 August 2026.

[2] Alene M Assemie MA Yismaw L Gedif G Ketema DB Gietaneh W Patient delay in the diagnosis of tuberculosis in Ethiopia: a systematic review and meta-analysis. BMC Infect Dis. 2020;20:797. 10.1186/s12879-020-05524-3 33109110 PMC7590610

[3] Barnabishvili M Ulrichs T Waldherr R Role of acceptability barriers in delayed diagnosis of Tuberculosis: Literature review from high burden countries. Acta Trop. 2016;161:106–13. 10.1016/j.actatropica.2016.06.014 27311390

[4] Bello S Afolabi RF Ajayi DT Sharma T Owoeye DO Oduyoye O Empirical evidence of delays in diagnosis and treatment of pulmonary tuberculosis: systematic review and meta-regression analysis. BMC Public Health. 2019;19:820. 10.1186/s12889-019-7026-4 31238906 PMC6593585

[5] Cai J Wang X Ma A Wang Q Han X Li Y Factors associated with patient and provider delays for tuberculosis diagnosis and treatment in Asia: a systematic review and meta-analysis. PLoS One. 2015;10:e0120088. 10.1371/journal.pone.0120088 25807385 PMC4373856

[6] de Vries SG Cremers AL Heuvelings CC Greve PF Visser BJ Belard S Barriers and facilitators to the uptake of tuberculosis diagnostic and treatment services by hard-to-reach populations in countries of low and medium tuberculosis incidence: a systematic review of qualitative literature. Lancet Infect Dis. 2017;17:e128–43. 10.1016/S1473-3099(16)30531-X 28291721

[7] Divala TH Lewis J Bulterys MA Lutje V Corbett EL Schumacher SG Missed opportunities for diagnosis and treatment in patients with TB symptoms: a systematic review. Public Health Action. 2022;12:10–7. 10.5588/pha.21.0022 35317535 PMC8908873

[8] Eltayeb D Pietersen E Engel M Abdullahi L Factors associated with tuberculosis diagnosis and treatment delays in Middle East and North Africa: a systematic review. East Mediterr Health J. 2020;26:477–86. 10.26719/2020.26.4.477 32338367

[9] Finnie RK Khoza LB van den Borne B Mabunda T Abotchie P Mullen PD Factors associated with patient and health care system delay in diagnosis and treatment for TB in sub-Saharan African countries with high burdens of TB and HIV. Trop Med Int Health. 2011;16:394–411. 10.1111/j.1365-3156.2010.02718.x 21320240

[10] Gamtesa DF Tola HH Mehamed Z Tesfaye E Alemu A Health care seeking behavior among presumptive tuberculosis patients in Ethiopia: a systematic review and meta-analysis. BMC Health Serv Res. 2020;20:445. 10.1186/s12913-020-05284-5 32429988 PMC7238571

[11] Getnet F Demissie M Assefa N Mengistie B Worku A Delay in diagnosis of pulmonary tuberculosis in low-and middle-income settings: systematic review and meta-analysis. BMC Pulm Med. 2017;17:202. 10.1186/s12890-017-0551-y 29237451 PMC5729407

[12] Grace SG Barriers to the implementation of isoniazid preventive therapy for tuberculosis in children in endemic settings: A review. J Paediatr Child Health. 2019;55:278–84. 10.1111/jpc.14359 30604557

[13] Jhaveri TA Jhaveri D Galivanche A Lubeck-Schricker M Voehler D Chung M Barriers to engagement in the care cascade for tuberculosis disease in India: A systematic review of quantitative studies. PLoS Med. 2024;21:e1004409. 10.1371/journal.pmed.1004409 38805509 PMC11166313

[14] Jiang Y, Cao B, Ohmagari N, Wu AH, Liu YX, Guo LP. Comprehensive understanding of health-seeking behaviour among pulmonary tuberculosis patients in China. int j tuberc lung dis. 2017;21(10):1094–9. 10.5588/ijtld.17.0227 28911351

[15] Krishnan L Akande T Shankar AV McIntire KN Gounder CR Gupta A Gender-related barriers and delays in accessing tuberculosis diagnostic and treatment services: a systematic review of qualitative studies. Tuberc Res Treat. 2014;2014:215059. 10.1155/2014/215059 24900921 PMC4037602

[16] Li Y Ehiri J Tang S Li D Bian Y Lin H Factors associated with patient, and diagnostic delays in Chinese TB patients: a systematic review and meta-analysis. BMC Med. 2013;11:156. 10.1186/1741-7015-11-156 23819847 PMC3699418

[17] Müller P Velez Lapao L Mixed methods systematic review and metasummary about barriers and facilitators for the implementation of cotrimoxazole and isoniazid-Preventive therapies for people living with HIV. PLoS One. 2022;17:e0251612. 10.1371/journal.pone.0251612 35231047 PMC8887777

[18] Obsa MS Daga WB Wosene NG Gebremedhin TD Edosa DC Dedecho AT Treatment seeking delay and associated factors among tuberculosis patients attending health facility in Ethiopia from 2000 to 2020: A systematic review and meta analysis. PLoS One. 2021;16:e0253746. 10.1371/journal.pone.0253746 34197515 PMC8248725

[19] Rutherford ME Hill PC Triasih R Sinfield R van Crevel R Graham SM Preventive therapy in children exposed to Mycobacterium tuberculosis: problems and solutions. Trop Med Int Health. 2012;17:1264–73. 10.1111/j.1365-3156.2012.03053.x 22862994

[20] Samal J Health Seeking Behaviour among Tuberculosis Patients in India: A Systematic Review. J Clin Diagn Res. 2016;10:LE01–6. 10.7860/JCDR/2016/19678.8598 27891362 PMC5121700

[21] Sreeramareddy CT Panduru KV Menten J Van den Ende J Time delays in diagnosis of pulmonary tuberculosis: a systematic review of literature. BMC Infect Dis. 2009;9:91. 10.1186/1471-2334-9-91 19519917 PMC2702369

[22] Sreeramareddy CT Qin ZZ Satyanarayana S Subbaraman R Pai M Delays in diagnosis and treatment of pulmonary tuberculosis in India: a systematic review. Int J Tuberc Lung Dis. 2014;18:255–66. 10.5588/ijtld.13.0585 24670558 PMC4070850

[23] Storla DG Yimer S Bjune GA A systematic review of delay in the diagnosis and treatment of tuberculosis. BMC Public Health. 2008;8:15. 10.1186/1471-2458-8-15 18194573 PMC2265684

[24] Sullivan BJ Esmaili BE Cunningham CK Barriers to initiating tuberculosis treatment in sub-Saharan Africa: a systematic review focused on children and youth. Glob Health Action. 2017;10:1290317. 10.1080/16549716.2017.1290317 28598771 PMC5496082

[25] Teibo TKA Andrade RLP Rosa RJ de Abreu PD Olayemi OA Alves YM Barriers That Interfere with Access to Tuberculosis Diagnosis and Treatment across Countries Globally: A Systematic Review. ACS Infect Dis. 2024;10:2600–14. 10.1021/acsinfecdis.4c00466 39023509 PMC11320565

[26] Teo AKJ Singh SR Prem K Hsu LY Yi S Duration and determinants of delayed tuberculosis diagnosis and treatment in high-burden countries: a mixed-methods systematic review and meta-analysis. Respir Res. 2021;22:251. 10.1186/s12931-021-01841-6 34556113 PMC8459488

[27] Varughese M Heffernan C Li MY Long R Time to diagnosis and treatment of pulmonary tuberculosis in indigenous peoples: a systematic review. BMC Infect Dis. 2023;23:131. 10.1186/s12879-023-08098-y 36882707 PMC9989566

[28] Yasobant S Bhavsar P Kalpana P Memon F Trivedi P Saxena D Contributing Factors in the Tuberculosis Care Cascade in India: A Systematic Literature Review. Risk Manag Healthc Policy. 2021;14:3275–86. 10.2147/RMHP.S322143 34408513 PMC8364383

[29] Kruk ME Gage AD Arsenault C Jordan K Leslie HH Roder-DeWan S High-quality health systems in the Sustainable Development Goals era: time for a revolution. Lancet Glob Health. 2018;6:e1196–252. 10.1016/S2214-109X(18)30386-3 30196093 PMC7734391

[30] Kruk ME Lewis TP Introduction to The Lancet Global Health’s Series on the People’s Voice Survey on Health System Performance. Lancet Glob Health. 2024;12:e14–5. 10.1016/S2214-109X(23)00512-0 38096886

[31] Pollock D Evans C Menghao Jia R Alexander L Pieper D Brandao de Moraes E “How-to”: scoping review? J Clin Epidemiol. 2024;176:111572. 10.1016/j.jclinepi.2024.111572 39426499

[32] Tricco AC Lillie E Zarin W O’Brien KK Colquhoun H Levac D PRISMA Extension for Scoping Reviews (PRISMA-ScR): Checklist and Explanation. Ann Intern Med. 2018;169:467–73. 10.7326/M18-0850 30178033

[33] Albert H Nathavitharana RR Isaacs C Pai M Denkinger CM Boehme CC Development, roll-out and impact of Xpert MTB/RIF for tuberculosis: what lessons have we learnt and how can we do better? Eur Respir J. 2016;48:516–25. 10.1183/13993003.00543-2016 27418550 PMC4967565

[34] World Health Organization. WHO global lists of high burden countries for TB, multidrug/rifampicin-resistant TB (MDR/RR-TB) and TB/HIV, 2021–2025. Geneva, Switzerland: World Health Organization; 2021. Available: https://iris.who.int/server/api/core/bitstreams/69a887d5-9f87-474a-b3ea-3b4f78a504e6/content. Accessed: 18 August 2026.

[35] Michie S van Stralen MM West R The behaviour change wheel: a new method for characterising and designing behaviour change interventions. Implement Sci. 2011;6:42. 10.1186/1748-5908-6-42 21513547 PMC3096582

[36] Bhering M Dalcolmo M Sarubbi V Junior Kritski A Barriers faced by patients in the diagnosis of multidrug-resistant tuberculosis in Brazil. Rev Saude Publica. 2022;56:60. 10.11606/s1518-8787.2022056004154 35766789 PMC9239425

[37] Asemahagn MA Alene GD Yimer SA A Qualitative Insight into Barriers to Tuberculosis Case Detection in East Gojjam Zone, Ethiopia. Am J Trop Med Hyg. 2020;103:1455–65. 10.4269/ajtmh.20-0050 32748766 PMC7543819

[38] Jarrett BA Woznica DM Tilchin C Mpungose N Motlhaoleng K Golub JE Promoting Tuberculosis Preventive Therapy for People Living with HIV in South Africa: Interventions Hindered by Complicated Clinical Guidelines and Imbalanced Patient-Provider Dynamics. AIDS Behav. 2020;24:1106–17. 10.1007/s10461-019-02675-6 31549265 PMC7085432

[39] Skinner D Claassens M It’s complicated: why do tuberculosis patients not initiate or stay adherent to treatment? A qualitative study from South Africa. BMC Infect Dis. 2016;16:712. 10.1186/s12879-016-2054-5 27887646 PMC5124309

[40] Mavhu W Dauya E Bandason T Munyati S Cowan FM Hart G Chronic cough and its association with TB-HIV co-infection: factors affecting help-seeking behaviour in Harare, Zimbabwe. Trop Med Int Health. 2010;15:574–9. 10.1111/j.1365-3156.2010.02493.x 20214762 PMC3374845

[41] Atre S Jagtap J Faqih M Dumbare Y Sawant T Ambike S Addressing patients’ unmet needs related to multidrug-resistant tuberculosis (MDR-TB) care: A qualitative research study from Pune city, India. PLoS One. 2023;18:e0295508. 10.1371/journal.pone.0295508 38153918 PMC10754455

[42] Chilala SB Silumbwe A Zulu JM Tetui M Bulawayo M Chewe M Individual, community and health systems factors influencing time to notification of tuberculosis: situating software and hardware bottlenecks in local health systems. BMC Health Serv Res. 2024;24:1241. 10.1186/s12913-024-11697-3 39415167 PMC11481775

[43] Oga-Omenka C Boffa J Kuye J Dakum P Menzies D Zarowsky C Understanding the gaps in DR-TB care cascade in Nigeria: A sequential mixed-method study. J Clin Tuberc Other Mycobact Dis. 2020;21:100193. 10.1016/j.jctube.2020.100193 33102811 PMC7578750

[44] Dahab R Sakellariou D Barriers to Accessing Maternal Care in Low Income Countries in Africa: A Systematic Review. Int J Environ Res Public Health. 2020;17:4292. 10.3390/ijerph17124292 32560132 PMC7344902

[45] Kassa MD Grace JM Noncommunicable Diseases Prevention Policies and Their Implementation in Africa: A Systematic Review. Public Health Rev. 2021;42:1604310. 10.3389/phrs.2021.1604310 35295954 PMC8865333

[46] Ojukwu E Pashaei A Maia JC Omobhude OF Tawfik A Nguyen Y Evaluating the impact of COVID-19 on the HIV care continuum across global income levels: a mixed-methods systematic review. AIDS Res Ther. 2025;22:115. 10.1186/s12981-025-00778-w 41152945 PMC12560595

[47] Ricci F Social implications of malaria and their relationships with poverty. Mediterr J Hematol Infect Dis. 2012;4:e2012048. 10.4084/mjhid.2012.048 22973492 PMC3435125

[48] Mukhalalati BA Taylor A Adult Learning Theories in Context: A Quick Guide for Healthcare Professional Educators. J Med Educ Curric Dev. 2019;6:2382120519840332. 10.1177/2382120519840332 31008257 PMC6458658

[49] Manzi A Hirschhorn LR Sherr K Chirwa C Baynes C Awoonor-Williams JK Mentorship and coaching to support strengthening healthcare systems: lessons learned across the five Population Health Implementation and Training partnership projects in sub-Saharan Africa. BMC Health Serv Res. 2017;17 Suppl 3:831. 10.1186/s12913-017-2656-7 29297323 PMC5763487

[50] Feyissa GT Balabanova D Woldie M How Effective are Mentoring Programs for Improving Health Worker Competence and Institutional Performance in Africa? A Systematic Review of Quantitative Evidence. J Multidiscip Healthc. 2019;12:989–1005. 10.2147/JMDH.S228951 31824166 PMC6901118

[51] Leslie HH Gage A Nsona H Hirschhorn LR Kruk ME Training And Supervision Did Not Meaningfully Improve Quality Of Care For Pregnant Women Or Sick Children In Sub-Saharan Africa. Health Aff (Millwood). 2016;35:1716–24. 10.1377/hlthaff.2016.0261 27605655

[52] Curran GM Bauer M Mittman B Pyne JM Stetler C Effectiveness-implementation hybrid designs: combining elements of clinical effectiveness and implementation research to enhance public health impact. Med Care. 2012;50:217–26. 10.1097/MLR.0b013e3182408812 22310560 PMC3731143

