# Supplementary material for: Frontline healthcare worker barriers impacting tuberculosis service demand and access in high burden settings: a scoping review
Source: J Glob Health. 2026 Sep 18;16:04243. doi: 10.7189/jogh.16.04243 (PMC13587593; doi:10.7189/jogh.16.04243)
Supplement: Supplementary file 1 [file jogh-16-04243-s001.pdf]

Supplement to: Cox SR, Hanrahan CF, Moe AH, Shearer K, Christian C, Sethi S, Hudson M, Ueshima M, Singh K, Dawson J, Angami K, Kubai N, Namasivayam M, Shete P, Golub JE, Kerkhoff AD. Frontline healthcare worker barriers impacting tuberculosis service demand and access in high burden settings: a scoping review. J Glob Health. 2026;16:04243.

Appendix S1: study protocol & search terms

Healthcare worker-related barriers to patient access to and demand for TB-related services: a scoping review

Protocol version: 1.0 dated 11 April 2025

Funder: Johnson & Johnson

Johns Hopkins University: Kate Shearer, Samyra R. Cox, Aye Hnin Moe, Sejal Sethi, Colleen Hanrahan, Jonathan E. Golub

UCSF: Canice Christian, Mollie Hudson, Andrew Kerkhoff, Priya Shete,

Background

Tuberculosis (TB) is the leading infectious cause of death worldwide. In 2023, there were an estimated 10.8 million people who developed active TB disease and 1.25 million deaths.[1] There is extensive literature published on health-seeking behavior and barriers related to uptake of TB preventive treatment, TB screening, TB diagnosis, and TB treatment initiation, with numerous systematic reviews published on these and related topics.[2–28] However, the existing reviews focus primarily on patient-level and health systems-level barriers to TB-related care. Yet, healthcare workers (HCWs) play a pivotal role in determining patient access to TB services. Healthcare workers' knowledge gaps, attitudes, practices, and competing priorities, among others factors, can create barriers that limit patients' access to TB services. Understanding these provider-side barriers is essential for developing effective interventions to improve patient access to TB services, which may lead to greater uptake of TB preventive treatment, improved TB diagnostic and treatment outcomes, and long-term reductions in TB incidence. Thus, this scoping review aims to summarize the literature on healthcare provider-related barriers that may limit patient access to and/or demand for TB-related services (i.e., prevention, diagnosis, treatment initiation, treatment completion) in high TB burden countries.

Methods

We will follow the Joanna Briggs Institute (JBI) guidance for scoping reviews and will report findings as per the Preferred Reporting Items for Systematic Reviews and Meta-Analyses extension for Scoping Reviews (PRISMA-ScR).

Inclusion criteria

Studies will be included if they describe healthcare provider-related barriers that limit patient access to and/or demand for TB-related services. Studies published between 2010-2025, in English, and reporting on original research findings from the World Health Organization's high-TB burden countries (TB, TB/HIV or drug-resistant TB) will be included.[31]

Exclusion criteria

We will exclude papers that do not report original research (i.e. protocol papers, systematic reviews, editorials, and/or commentaries), that are abstracts or conference proceedings only, not available in English, or if we are unable to locate the paper. We will exclude papers published prior to 2010 when major diagnostic advancements like Xpert MTB/RIF were introduced, which may have reduced healthcare worker-related barriers to TB treatment initiation, in particular, due to improved diagnostic accuracy.

Definitions

Healthcare provider	Individuals who provide medical care or treatment for TB—which can include consultation, providing medication, or choosing appropriate TB prevention and/or treatment regimens—and may or may not have formal medical qualifications
Health-seeking	How individuals who are ill and/or with presumptive TB seek medical care from health facilities for their symptoms
Access	The opportunity to obtain health care services, including the ability to perceive, seek, reach, pay, and engage with TB-related care
Demand	Efforts employed to create, increase uptake, and sustain demand of TB services (i.e. diagnostic tests, screening, treatment)<br><br>Strategies that promote awareness, understanding, and uptake of or retention in TB prevention, screening, diagnosis, treatment, and post-treatment services across the care cascade
Barrier	Factors, conditions, or circumstances that impede, restrict, or prevent healthcare providers from effectively delivering—or patients from successfully accessing and utilizing—tuberculosis prevention, screening, diagnosis, and treatment services across the care cascade. Barriers may be structural, organizational, cultural, knowledge-based, attitudinal, or behavioral in nature, and may affect one or multiple points along the TB care pathway.
TB-related services	TB preventive treatment, TB diagnosis, TB treatment (initiation/adherence/completion)
High TB burden countries	As defined by the World Health Organization (2021-2025)<br><br>30 high TB burden countries: The 20 countries with the highest estimated numbers of incident TB cases, plus the top 10 countries with the highest estimated TB incidence rate that are not in the top 20 by absolute number (threshold, >10 000 estimated incident TB cases per year).<br><br>30 high TB/HIV burden countries: The 20 countries with the highest estimated numbers of incident TB cases among people living with HIV, plus the top 10 countries with the highest estimated TB/HIV incidence rate that are not in the top 20 by absolute number (threshold, >1000 estimated incident TB/HIV cases per year).<br><br>30 high MDR/RR-TB burden countries: The 20 countries with the highest estimated numbers of incident MDR/RR-TB cases, plus the top 10 countries with the highest estimated MDR/RR-TB incidence rate that are not in the top 20 by absolute number (threshold, >1000 estimated incident MDR/RR-TB cases per year).

Selection of papers

We will search PubMed using search terms covering the concepts of TB, high-burden countries, barriers, and healthcare workers. A complete list of search terms is included as an appendix to the study protocol. The resulting titles and abstracts will be uploaded into Covidence and duplicates will be removed. Two independent reviewers will screen the titles and abstracts of the remaining search results to determine if they should be included in the full text review. Any conflicts will be resolved by a third reviewer. The selected full text articles will then be reviewed by both independent reviewers to determine final inclusion/exclusion status. Conflicts will again be resolved by a third reviewer. Any paper that is deemed ineligible for inclusion will be recorded as rejected with a reason specified in Covidence. Finally, we will search the reference list of each included paper for any additional articles not identified by our original PubMed search. Review of additional papers will follow the same process above. Results of this screening process will be presented using a flow diagram.

Data abstraction

Results from each of the final included papers will be abstracted using a digital form including the title, authors, year of publication, and location, the study type (qualitative, quantitative, mixed-methods), the TB service(s) (prevention, screening, diagnosis, treatment initiation/completion), TB type(s) (drug-sensitive, drug-resistant, both), healthcare worker characteristics (nurses, doctors, community health workers, other), patient population the healthcare workers provide services for (adults, children, people living with HIV, household contacts, other), health provider-related barriers identified, and the theory or theories used to map or characterize barriers, if any. The first five papers will be reviewed by both reviewers to check for consistency with data abstracted independently thereafter.

Data Synthesis

Data will be organized using a implementation science framework of healthcare provider-related barriers with findings presented narratively with supporting tables and figures. We will also geographically map identified barriers across high-burden countries to visualize differences across settings and will summarize barriers at each step of the TB care cascade (prevention, screening, diagnosis, treatment) to highlight differences across cascade steps. Finally, we will identify research gaps and priority areas for intervention and strategy development.

Bias assessment

We will not perform a risk of bias assessment as this is not a standard component of a scoping review.

Study Protocol References

1. World Health Organization. *Global tuberculosis report 2024*. https://www.who.int/publications/i/item/9789240101531 (2024).
2. Alene, M., Assemie, M. A., Yismaw, L., *et al.* Patient delay in the diagnosis of tuberculosis in Ethiopia: a systematic review and meta-analysis. *BMC Infect Dis* **20**, (2020).
3. Barnabishvili, M., Ulrichs, T. & Waldherr, R. Role of acceptability barriers in delayed diagnosis of Tuberculosis: Literature review from high burden countries. *Acta Tropica* vol. 161 106–113 (2016).
4. Bello, S., Afolabi, R. F., Ajayi, D. T., *et al.* Empirical evidence of delays in diagnosis and treatment of pulmonary tuberculosis: Systematic review and meta-regression analysis. *BMC Public Health* vol. 19 (2019).
5. Cai, J., Wang, X., Ma, A., *et al.* Factors associated with patient and provider delays for tuberculosis diagnosis and treatment in Asia: A systematic review and meta-analysis. *PLoS One* **10**, (2015).
6. de Vries, S. G., Cremers, A. L., Heuvelings, C. C., *et al.* Barriers and facilitators to the uptake of tuberculosis diagnostic and treatment services by hard-to-reach populations in countries of low and medium tuberculosis incidence: a systematic review of qualitative literature. *The Lancet Infectious Diseases* vol. 17 e128–e143 (2017).
7. Divala, T. H., Lewis, J., Bulterys, M. A., *et al.* Missed opportunities for diagnosis and treatment in patients with TB symptoms: a systematic review. *Public Health Action* **12**, 10–17 (2022).
8. Eltayeb, D., Pietersen, E., Engel, M., *et al.* Factors associated with tuberculosis diagnosis and treatment delays in middle east and North Africa: A systematic review. *Eastern Mediterranean Health Journal* **26**, 477–487 (2020).
9. Finnie, R. K. C., Khoza, L. B., van den Borne, B., *et al.* Revue systématique: Facteurs associés au patient et au système de santé dans le retard du diagnostic et du traitement de la tuberculose dans les pays d'Afrique subsaharienne à forte charge de TB et VIH. *Tropical Medicine and International Health* vol. 16 394–411 (2011).
10. Gamtesa, D. F., Tola, H. H., Mehamed, Z., *et al.* Health care seeking behavior among presumptive tuberculosis patients in Ethiopia: A systematic review and meta-analysis. *BMC Health Serv Res* **20**, (2020).
11. Getnet, F., Demissie, M., Assefa, N., *et al.* Delay in diagnosis of pulmonary tuberculosis in low- and middle-income settings: Systematic review and meta-analysis. *BMC Pulm Med* **17**, (2017).
12. Jhaveri, T. A., Jhaveri, D., Galivanche, A., *et al.* Barriers to engagement in the care cascade for tuberculosis disease in India: A systematic review of quantitative studies. *PLoS Medicine* vol. 21 (2024).
13. Jiang, Y., Cao, B., Ohmagari, N., *et al.* Comprehensive understanding of health-seeking behaviour among pulmonary tuberculosis patients in China. *International Journal of Tuberculosis and Lung Disease* **21**, 1094–1099 (2017).
14. Krishnan, L., Akande, T., Shankar, A. V., *et al.* Gender-Related Barriers and Delays in Accessing Tuberculosis Diagnostic and Treatment Services: A Systematic Review of Qualitative Studies. *Tuberc Res Treat* **2014**, 1–14 (2014).
15. Li, Y., Ehiri, J., Tang, S., *et al.* Factors associated with patient, and diagnostic delays in Chinese TB patients: A systematic review and meta-analysis. *BMC Med* **11**, (2013).
16. Obsa, M. S., Daga, W. B., Wosene, N. G., *et al.* Treatment seeking delay and associated factors among tuberculosis patients attending health facility in Ethiopia from 2000 to 2020: A systematic review and meta analysis. *PLoS ONE* vol. 16 (2021).
17. Samal, J. Health seeking behaviour among tuberculosis patients in India: A systematic review. *Journal of Clinical and Diagnostic Research* vol. 10 (2016).
18. Sreeramareddy, C. T., Panduru, K. V., Menten, J., *et al.* Time delays in diagnosis of pulmonary tuberculosis: A systematic review of literature. *BMC Infectious Diseases* vol. 9 (2009).

19. Sreeramareddy, C. T., Qin, Z. Z., Satyanarayana, S., *et al.* Delays in diagnosis and treatment of pulmonary tuberculosis in India: A systematic review. *International Journal of Tuberculosis and Lung Disease* **18**, 255–266 (2014).
20. Storla, D. G., Yimer, S. & Bjune, G. A. A systematic review of delay in the diagnosis and treatment of tuberculosis. *BMC Public Health* vol. 8 (2008).
21. Sullivan, B. J., Esmaili, B. E. & Cunningham, C. K. Barriers to initiating tuberculosis treatment in sub-Saharan Africa: A systematic review focused on children and youth. *Global Health Action* vol. 10 (2017).
22. Teibo, T. K. A., Andrade, R. L. de P., Rosa, R. J., *et al.* Barriers That Interfere with Access to Tuberculosis Diagnosis and Treatment across Countries Globally: A Systematic Review. *ACS Infectious Diseases* vol. 10 2600–2614 (2024).
23. Teo, A. K. J., Singh, S. R., Prem, K., *et al.* Duration and determinants of delayed tuberculosis diagnosis and treatment in high-burden countries: a mixed-methods systematic review and meta-analysis. *Respir Res* **22**, (2021).
24. Varughese, M., Heffernan, C., Li, M. Y., *et al.* Time to diagnosis and treatment of pulmonary tuberculosis in indigenous peoples: a systematic review. *BMC Infect Dis* **23**, (2023).
25. Yasobant, S., Bhavsar, P., Kalpana, P., *et al.* Contributing factors in the tuberculosis care cascade in india: A systematic literature review. *Risk Management and Healthcare Policy* vol. 14 3275–3286 (2021).
26. Müller, P. & Lapão, L. V. Mixed methods systematic review and metasummary about barriers and facilitators for the implementation of cotrimoxazole and isoniazid-Preventive therapies for people living with HIV. *PLoS One* **17**, (2022).
27. Rutherford, M. E., Hill, P. C., Triasih, R., *et al.* Preventive therapy in children exposed to Mycobacterium tuberculosis: Problems and solutions. *Tropical Medicine and International Health* vol. 17 1264–1273 (2012).
28. Grace, S. G. Barriers to the implementation of isoniazid preventive therapy for tuberculosis in children in endemic settings: A review. *Journal of Paediatrics and Child Health* vol. 55 278–284 (2019).
29. Pollock, D., Evans, C., Menghao Jia, R., *et al.* “How-to”: scoping review? *J Clin Epidemiol* **176**, 111572 (2024).
30. Tricco, A. C., Lillie, E., Zarin, W., *et al.* PRISMA extension for scoping reviews (PRISMA-ScR): Checklist and explanation. *Annals of Internal Medicine* vol. 169 467–473 (2018).
31. World Health Organization. *WHO global lists of high burden countries for tuberculosis (TB), TB/HIV and multidrug/rifampicin-resistant TB (MDR/RR-TB), 2021-2025*. (2021).
32. Albert, H., Nathavitharana, R. R., Isaacs, C., *et al.* Development, roll-out and impact of Xpert MTB/RIF for tuberculosis: What lessons have we learnt and how can we do better? *European Respiratory Journal* vol. 48 516–525 (2016).
33. Michie, S., van Stralen, M. M. & West, R. The behaviour change wheel: A new method for characterising and designing behaviour change interventions. *Implementation Science* **6**, (2011).
34. Bhering, M., Dalcolmo, M., Sarubbi, V., *et al.* Barriers faced by patients in the diagnosis of multidrug-resistant tuberculosis in Brazil. *Rev Saude Publica* **56**, (2022).
35. Asemahagn, M. A., Alene, G. D. & Yimer, S. A. A qualitative insight into barriers to tuberculosis case detection in east gojjam zone, Ethiopia. *American Journal of Tropical Medicine and Hygiene* **103**, 1455–1465 (2020).
36. Jarrett, B. A., Woznica, D. M., Tilchin, C., *et al.* Promoting tuberculosis preventive therapy for people living with HIV in South Africa: Interventions hindered by complicated clinical guidelines and imbalanced patient-provider dynamics. *AIDS Behav* **24**, 1106–1117 (2020).
37. Skinner, D. & Claassens, M. It’s complicated: Why do tuberculosis patients not initiate or stay adherent to treatment? A qualitative study from South Africa. *BMC Infect Dis* **16**, 712 (2016).

38. Mavhu, W., Dauya, E., Bandason, T., *et al.* Chronic cough and its association with TB-HIV co-infection: Factors affecting help-seeking behaviour in Harare, Zimbabwe. *Tropical Medicine and International Health* **15**, 574–579 (2010).
39. Mathew, G., Kumar, S. C. S., Cherian, K. M., *et al.* Revisions in TB programme - boon or bane? A qualitative study exploring barriers and facilitators among health care workers in private and public sector, Kerala. *Indian Journal of Tuberculosis* **68**, 356–362 (2021).
40. Oga-Omenka, C., Boffa, J., Kuye, J., *et al.* Understanding the gaps in DR-TB care cascade in Nigeria: A sequential mixed-method study. *J Clin Tuberc Other Mycobact Dis* **21**, 100193 (2020).
41. Atre, S., Jagtap, J., Faqih, M., *et al.* Addressing patients' unmet needs related to multidrug-resistant tuberculosis (MDR-TB) care: A qualitative research study from Pune city, India. *PLoS One* **18**, e0295508 (2023).
42. Chilala, S. B., Silumbwe, A., Zulu, J. M., *et al.* Individual, community and health systems factors influencing time to notification of tuberculosis: situating software and hardware bottlenecks in local health systems. *BMC Health Serv Res* **24**, 1241 (2024).

Study Protocol Appendix: PubMed Search Terms

Concept: TB

"Tuberculosis"[Mesh:NoExp] OR "tuberculosis" [tiab] OR tuberculos*[tiab] OR TB[tiab] OR Tuberculosis, Multidrug-Resistant[mesh] OR Multidrug-Resistant Tuberculosis[tiab] OR Multi-Drug Resistant Tuberculosis[tiab] OR MDR-TB[tiab] OR Extensively Drug-Resistant Tuberculosis[Mesh] OR Extensively Drug Resistant Tuberculosis[tiab] OR Extremely Drug-Resistant Tuberculosis[tiab] OR Extensively Drug-Resistant Tuberculoses[tiab] OR XDR-TB[tiab] OR Tuberculosis, Extrapulmonary[mesh] OR Extrapulmonary Tuberculos*[tiab] OR Extra Pulmonary Tuberculosis[tiab] OR EPTB[tiab] OR DS-TB[tiab] OR DS TB[tiab]

Concept: High burden countries (TB, TB-HIV, MDRTB)

angola[mh] OR azerbaijan[mh] OR bangladesh[mh] OR republic of belarus[mh] OR botswana[mh] OR brazil[mh] OR cameroon[mh] OR central african republic[mh] OR china[mh] OR democratic republic of the congo[mh] OR congo[mh] OR eswatini[mh] OR Ethiopia[mh] OR gabon[mh] OR guinea[mh] OR guinea-bissau[mh] OR india[mh] OR indonesia[mh] OR kazakhstan[mh] OR kenya[mh] OR democratic people's republic of korea[mh] OR kyrgyzstan[mh] OR lesotho[mh] OR liberia[mh] OR malawi[mh] OR moldova[mh] OR mongolia[mh] OR mozambique[mh] OR myanmar[mh] OR namibia[mh] OR nepal[mh] OR nigeria[mh] OR pakistan[mh] OR papua new guinea[mh] OR peru[mh] OR philippines[mh] OR russia[mh] OR sierra leone[mh] OR somalia[mh] OR south africa[mh] OR tajikistan[mh] OR thailand[mh] OR tanzania[mh] OR uganda[mh] OR ukraine[mh] OR uzbekistan[mh] OR Vietnam[mh] OR zambia[mh] OR zimbabwe[mh] OR angola[tiab] OR azerbaijan[tiab] OR bangladesh[tiab] OR republic of belarus[tiab] OR botswana[tiab] OR brazil[tiab] OR cameroon[tiab] OR central african republic[tiab] OR china[tiab] OR democratic republic of the congo[tiab] OR congo[tiab] OR eswatini[tiab] OR Ethiopia[tiab] OR gabon[tiab] OR guinea[tiab] OR guinea-bissau[tiab] OR india[tiab] OR indonesia[tiab] OR kazakhstan[tiab] OR kenya[tiab] OR democratic people's republic of korea[tiab] OR kyrgyzstan[tiab] OR lesotho[tiab] OR liberia[tiab] OR malawi[tiab] OR moldova[tiab] OR mongolia[tiab] OR mozambique[tiab] OR myanmar[tiab] OR namibia[tiab] OR nepal[tiab] OR nigeria[tiab] OR pakistan[tiab] OR papua new guinea[tiab] OR peru[tiab] OR philippines[tiab] OR russia[tiab] OR sierra leone[tiab] OR somalia[tiab] OR south africa[tiab] OR tajikistan[tiab] OR thailand[tiab] OR tanzania[tiab] OR uganda[tiab] OR ukraine[tiab] OR uzbekistan[tiab] OR Vietnam[tiab] OR zambia[tiab] OR zimbabwe[tiab]

Concept: Barriers

barrier*[tiab] OR challenge*[tiab] OR delay[tiab] OR obstacle*[tiab] OR problem* [tiab] OR factor* [tiab] OR gap [tiab] OR observation*[tiab] OR experience*[tiab] OR perception*[tiab] OR determinant*[tiab] OR implementation[tiab] OR provision[tiab]

Concept: Healthcare worker

"Health Personnel"[Mesh:NoExp] OR Community Health Workers[mesh] OR Health Worker*[tiab] OR Healthcare Worker*[tiab] OR Health Care Provider*[tiab] OR Provider*[tiab] OR Health Care Professional* [tiab] OR Physicians[mesh] OR Physician*[tiab] OR doctor*[tiab] OR nurses[mesh] OR nurs*[tiab] OR pharmacist*[tiab] OR clinic[tiab] OR hospital[tiab] OR facilit*[tiab]

Concept: Article type

Review [Publication Type]

Appendix S2: Thematic category definitions and considerations

We distinguished between knowledge and skills, and motivation as follows: knowledge and skills were related to barriers such as a lack of understanding or ability to provide TB services per guidelines; and motivation was related to barriers such as the amount of effort a FLHCW was willing to engage in to provide quality TB services. Some overlap is inherent to the barriers as categorized. For example, poor provider communication and attitudes may also be related to inadequate knowledge and skills; we analyzed these as mutually exclusive categories because issues with FLHCW communication and attitudes were frequently mentioned in the included papers. Similarly, the communication and attitudes category does not include use of stigmatizing or discriminatory language, which was analyzed separately because it arose as a clear theme.

Appendix S3: Complete list of high TB burden countries included in the review

The 30 high TB burden countries	The 30 high TB/HIV burden countries	The 30 high MDR-TB burden countries
The 20 countries with the highest estimated numbers of incident TB cases:<br><br>1. Angola<br>2. Bangladesh<br>3. Brazil<br>4. China<br>5. Democratic People's Republic of Korea<br>6. Democratic Republic of Congo<br>7. Ethiopia<br>8. India<br>9. Indonesia<br>10. Kenya<br>11. Mozambique<br>12. Myanmar<br>13. Nigeria<br>14. Pakistan<br>15. Philippines<br>16. South Africa<br>17. Thailand<br>18. Uganda<br>19. United Republic of Tanzania<br>20. Viet Nam<br><br>The additional 10 countries with the highest estimated TB incidence rate that are not in the top 20 by absolute number (threshold, >10 000 estimated incident TB cases per year):<br><br>21. Central African Republic<br>22. Congo<br>23. Gabon<br>24. Lesotho<br>25. Liberia<br>26. Mongolia<br>27. Namibia<br>28. Papua New Guinea<br>29. Sierra Leone<br>30. Zambia	The 20 countries with the highest estimated numbers of incident TB cases among people living with HIV:<br><br>1. Brazil<br>2. Cameroon<br>3. China<br>4. Democratic Republic of Congo<br>5. Ethiopia<br>6. India<br>7. Indonesia<br>8. Kenya<br>9. Malawi<br>10. Mozambique<br>11. Myanmar<br>12. Nigeria<br>13. Philippines<br>14. Russian Federation<br>15. South Africa<br>16. Thailand<br>17. Uganda<br>18. United Republic of Tanzania<br>19. Zambia<br>20. Zimbabwe<br><br>The additional 10 countries with the highest estimated TB/HIV incidence rate that are not in the top 20 by absolute number (threshold, >1000 estimated incident TB/HIV cases per year):<br><br>21. Boswana<br>22. Central African Republic<br>23. Congo<br>24. Eswatini<br>25. Gabon<br>26. Guinea<br>27. Guinea-Bissau<br>28. Lesotho<br>29. Liberia<br>30. Namibia	The 20 countries with the highest estimated numbers of incident MDR/RR-TB cases:<br><br>1. Angola<br>2. Bangladesh<br>3. China<br>4. Democratic People's Republic of Korea<br>5. Democratic Republic of Congo<br>6. India<br>7. Indonesia<br>8. Kazakhstan<br>9. Mozambique<br>10. Myanmar<br>11. Nigeria<br>12. Pakistan<br>13. Peru<br>14. Philippines<br>15. Russian Federation<br>16. Somalia<br>17. South Africa<br>18. Ukraine<br>19. Uzbekistan<br>20. Viet Nam<br><br>The additional 10 countries with the highest estimated MDR/RR-TB incidence rate that are not in the top 20 by absolute number (threshold, >1000 estimated incident MDR/RR-TB cases per year):<br><br>21. Azerbaijan<br>22. Belarus<br>23. Kyrgyzstan<br>24. Mongolia<br>25. Nepal<br>26. Papua New Guinea<br>27. Republic of Moldova<br>28. Tajikistan<br>29. Zambia<br>30. Zimbabwe

The countries in each category are listed in alphabetical order.
Abbreviations: TB=tuberculosis; HIV= human immunodeficiency virus; MDR=multi-drug resistant; RR=rifampicin resistant
Reference: WHO global lists of high burden countries for TB, multidrug/rifampicin-resistant TB (MDR/RR-TB) and TB/HIV, 2021–2025. Geneva: World Health Organization; 2021. Licence: CC BY-NC-SA 3.0 IGO.

Appendix S4: Complete list of included publications

#	Author & Year of publication	Country in which the study conducted	Care steps covered	Patient population	Patient risk factors
1	Abbas 2023	Pakistan	Treatment Completion/Adherence	Adult	None
2	AbbasAli 2024	India	Treatment Completion/Adherence	Adult	None
3	Abebe 2022	Ethiopia	Treatment Completion/Adherence	Not specified	None
4	Abraham 2024	Ethiopia	Active Case Finding	Not specified	None
5	Abubakar 2024	Pakistan	Treatment Completion/Adherence	Not specified	None
6	Achanta 2013	India	Diagnosis & Treatment Initiation<br>Treatment Completion/Adherence	Not specified	None
7	Adams 2017	South Africa	General TB care	Not specified	Mining
8	Ahmad 2024	India	Diagnosis & Treatment Initiation	Not specified	None
9	Ahmed 2021	South Africa	TB Preventive Treatment	Adult	None
10	Aibana 2020	Ukraine	Treatment Completion/Adherence	Adult	None
11	Ajudua 2022	South Africa	Active Case Finding	Not specified	None
12	Akamike 2022	Nigeria	TB Preventive Treatment	Not specified	PLWH
13	Ali 2019	Pakistan	Treatment Completion/Adherence	Not specified	None
14	Alvi 2024	India	TB Preventive Treatment	Pediatric<br>Adult	Household contacts
15	Amuge 2024	Uganda	TB Preventive Treatment	Pediatric	PLWH
16	Andom 2022	Lesotho	Diagnosis & Treatment Initiation	Pediatric<br>Adult	None
17	Arini 2022	Indonesia	General TB care	Not specified	Diabetes
18	Arscott-Mills 2017	Botswana	TB Preventive Treatment<br>Diagnosis & Treatment Initiation	Pediatric	None
19	ArtawanEka Putra 2013	Indonesia	Diagnosis & Treatment Initiation	Not specified	None
20	Asemahagn 2020	Ethiopia	Active Case Finding	Not specified	None
21	Atif 2016	Pakistan	Diagnosis & Treatment Initiation<br>Treatment Completion/Adherence	Adult	None

22	Atkins 2011	South Africa	Diagnosis & Treatment Initiation<br>Treatment Completion/Adherence	Not specified	None
23	Atre 2023	India	General TB care	Adult	None
24	Ayakaka 2017	Uganda	Active Case Finding	Not specified	Household contacts
25	Baloyi 2022	South Africa	TB Preventive Treatment	Pediatric<br>Adult	None
26	Baloyi 2024	South Africa	General TB care	Pediatric	None
27	Baniqued 2020	Philippines	Treatment Completion/Adherence	Adult	None
28	Belgaumkar 2018	India	TB Preventive Treatment<br>Active Case Finding	Pediatric	Household contacts
29	Bhagat 2011	India	Treatment Completion/Adherence	Not specified	None
30	Bhering 2022	Brazil	Diagnosis & Treatment Initiation	Adult	None
31	Bieh 2017	Nigeria	General TB care	Adult	None
32	Bjerrum 2012	Tanzania	Diagnosis & Treatment Initiation	Pediatric	None
33	Boffa 2021	South Africa	Diagnosis & Treatment Initiation	Adult	None
34	Brady 2018	Angola	Diagnosis & Treatment Initiation<br>Treatment Completion/Adherence<br>General TB care	Adult	Migrant workers
35	Bulage 2014	Uganda	General TB care	Adult	None
36	Catalani 2014	Kenya	TB Preventive Treatment<br>Diagnosis & Treatment Initiation	Not specified	PLWH
37	Cattamanchi 2015	Uganda	Diagnosis & Treatment Initiation<br>General TB care	Not specified	None
38	Caturegli 2020	South Africa	TB Preventive Treatment	Adult	PLWH
39	Chawla 2021	India	Diagnosis & Treatment Initiation	Pediatric	Household contacts
40	Chetty-Makkan 2021	South Africa	Active Case Finding<br>General TB care	Not specified	None
41	Chiang 2015	Peru	Diagnosis & Treatment Initiation	Pediatric	Household contacts
42	Chiang 2017	Peru	Diagnosis & Treatment Initiation	Pediatric	None
43	Chihota 2015	South Africa	Diagnosis & Treatment Initiation	Adult	None
44	Chijioke-Akaniro 2023	Nigeria	Diagnosis & Treatment Initiation	Pediatric<br>Adult	None

			Treatment Completion/Adherence		
45	Chilala 2024	Zambia	Diagnosis & Treatment Initiation	Adult	None
46	Chimbatata NBW 2017	Malawi	Diagnosis & Treatment Initiation	Not specified	None
47	Christian 2024	Uganda	TB Preventive Treatment	Adult	PLWH
48	Colvin 2019	Philippines	TB Preventive Treatment<br>Treatment Completion/Adherence<br>General TB care	Adult	None
49	Cowan 2013	Ethiopia	TB Preventive Treatment<br>General TB care	Not specified	None
50	Dabaro 2017	Ethiopia	Diagnosis & Treatment Initiation	Not specified	None
51	Daboer 2013	Nigeria	General TB care	Not specified	None
52	Daftary 2014	South Africa	Diagnosis & Treatment Initiation<br>Treatment Completion/Adherence<br>General TB care	Adult	PLWH
53	Daftary 2016	South Africa	General TB care	Not specified	PLWH
54	daSilva 2017	Brazil	Treatment Completion/Adherence	Adult	None
55	Deshmukh 2015	India	Treatment Completion/Adherence	Adult	None
56	Elbireer 2011	Uganda	Treatment Completion/Adherence	Adult	PLWH
57	Endo 2022	Philippines	Treatment Completion/Adherence	Not specified	None
58	Feyisa 2022	Ethiopia	Diagnosis & Treatment Initiation	Not specified	None
59	Foster 2023	South Africa	General TB care	Pediatric<br>Adult	PLWH
60	Garg 2020	India	Diagnosis & Treatment Initiation	Not specified	None
61	Gayathri 2020	India	Diagnosis & Treatment Initiation	Not specified	None
62	Geadas 2024	Peru	Diagnosis & Treatment Initiation	Adult	None
63	Gebremariam 2010	Ethiopia	Treatment Completion/Adherence	Not specified	None
64	Getnet 2017	Ethiopia	Active Case Finding<br>Diagnosis & Treatment Initiation	Adult	None
65	GugssaBoru 2017	Ethiopia	Treatment Completion/Adherence	Adult	None
66	Haldane 2024	China	Diagnosis & Treatment Initiation	Not specified	None
67	Hasker 2010	Uzbekistan	Treatment Completion/Adherence	Not specified	None

68	Hayward 2024	South Africa	General TB care	Pediatric Adult	None
69	Ibrahim 2014	Nigeria	Treatment Completion/Adherence	Adult	None
70	Ibrahim 2014	Nigeria	Treatment Completion/Adherence	Not specified	None
71	Izudi 2019	Uganda	General TB care	Not specified	None
72	Jacobson 2017	South Africa	TB Preventive Treatment	Adult	PLWH
73	Jaramillo 2023	Philippines	General TB care	Not specified	None
74	Jarrett 2020	South Africa	TB Preventive Treatment	Adult	PLWH
75	Kalema 2021	Uganda	TB Preventive Treatment	Adult	PLWH
76	Kallon 2022	South Africa	General TB care	Adult	PLWH
77	Katende 2021	Uganda	General TB care	Adult	PLWH
78	Kaumba 2024	Zambia	TB Preventive Treatment Diagnosis & Treatment Initiation	Pediatric	None
79	Khan 2016	Pakistan	Treatment Completion/Adherence	Not specified	None
80	Kigozi 2020	South Africa	Active Case Finding	Not specified	None
81	KS 2018	India	TB Preventive Treatment Diagnosis & Treatment Initiation	Pediatric	None
82	Kumar 2025	India	TB Preventive Treatment	Pediatric	Household contacts
83	Kumwenda 2016	Malawi	General TB care	Not specified	None
84	Kumwenda 2017	Malawi	Diagnosis & Treatment Initiation	Adult	Pregnancy
85	Laghari 2021	Pakistan	Treatment Completion/Adherence	Pediatric	None
86	Lai 2019	Ethiopia	TB Preventive Treatment	Not specified	PLWH
87	Lajore 2024	Ethiopia	General TB care	Not specified	None
88	Lester 2010	South Africa	TB Preventive Treatment	Adult	PLWH
89	Li 2014	China	General TB care	Adult	None
90	Loveday 2019	South Africa	Diagnosis & Treatment Initiation Treatment Completion/Adherence	Adult	Pregnancy
91	Loveday 2020	South Africa	Diagnosis & Treatment Initiation Treatment Completion/Adherence	Adult	Pregnancy
92	Lyakurwa 2021	Tanzania	Treatment Completion/Adherence	Adult	None

93	Mabunda 2016	South Africa	Treatment Completion/Adherence	Not specified	None
94	Mabuza 2018	South Africa	General TB care	Adult	PLWH
95	Mala 2014	Ethiopia	Diagnosis & Treatment Initiation	Adult	None
96	Mandera 2024	Uganda	Diagnosis & Treatment Initiation	Pediatric	None
97	Marahatta 2020	Nepal	Diagnosis & Treatment Initiation<br>Treatment Completion/Adherence	Not specified	None
98	Marais 2019	South Africa	TB Preventive Treatment<br>Diagnosis & Treatment Initiation	Not specified	None
99	Marangu 2017	Kenya	Diagnosis & Treatment Initiation	Not specified	None
100	Matakanye 2019	South Africa	Diagnosis & Treatment Initiation<br>Treatment Completion/Adherence	Not specified	None
101	Mathew 2021	India	General TB care	Pediatric<br>Adult	None
102	Mavhu 2010	Zimbabwe	Diagnosis & Treatment Initiation	Adult	None
103	McDowell 2016	India	Diagnosis & Treatment Initiation	Not specified	None
104	McNally 2019	Peru	Diagnosis & Treatment Initiation<br>Treatment Completion/Adherence	Adult	None
105	Megerso 2020	Ethiopia	Diagnosis & Treatment Initiation<br>Treatment Completion/Adherence	Adult	None
106	Melgar 2021	Zambia	TB Preventive Treatment	Pediatric<br>Adult	PLWH
107	Mercaldo 2021	Uganda	Diagnosis & Treatment Initiation	Adult	None
108	Mindachew 2014	Ethiopia	TB Preventive Treatment	Adult	PLWH
109	Mnyambwa 2021	Other: Tanzania, Kenya, and Uganda	Active Case Finding<br>Diagnosis & Treatment Initiation	Pediatric<br>Adult	PLWH
110	Moodley 2020	South Africa	Treatment Completion/Adherence	Adult	None
111	Moolphate 2013	Thailand	TB Preventive Treatment	Not specified	PLWH
112	Mpagama 2019	Tanzania	Diagnosis & Treatment Initiation	Not specified	PLWH
113	Mpagama 2020	Tanzania	Diagnosis & Treatment Initiation<br>General TB care	Adult	None

114	Mphothulo 2025	South Africa	Diagnosis & Treatment Initiation<br>Treatment Completion/Adherence	Adult	None
115	Mukerji 2020	India	Diagnosis & Treatment Initiation<br>Treatment Completion/Adherence	Adult	None
116	Mulaku 2023	Kenya	Diagnosis & Treatment Initiation	Adult	None
117	Mundra 2019	India	Diagnosis & Treatment Initiation	Not specified	None
118	Murdoch 2021	South Africa	Diagnosis & Treatment Initiation<br>General TB care	Not specified	None
119	Mussie 2021	Ethiopia	General TB care	Not specified	None
120	Mustafa 2018	Pakistan	Diagnosis & Treatment Initiation	Not specified	None
121	Muttamba 2021	Uganda	Diagnosis & Treatment Initiation<br>General TB care	Pediatric	None
122	Nagarajan 2022	India	General TB care	Adult	None
123	Naidoo 2015	South Africa	Diagnosis & Treatment Initiation	Adult	None
124	Nakaggwa 2016	Uganda	Treatment Completion/Adherence	Pediatric<br>Adult	PLWH<br>Prior TB disease
125	Nansera 2010	Uganda	Diagnosis & Treatment Initiation	Not specified	PLWH
126	Newtonraj 2019	India	Diagnosis & Treatment Initiation	Pediatric<br>Adult	PLWH
127	Nezenega 2013	Ethiopia	Treatment Completion/Adherence	Pediatric<br>Adult	None
128	Nóbrega 2010	Brazil	Active Case Finding<br>Diagnosis & Treatment Initiation	Not specified	None
129	Oga-Omenka 2020	Nigeria	Diagnosis & Treatment Initiation<br>Treatment Completion/Adherence	Pediatric<br>Adult	None
130	Oga-Omenka 2020	Nigeria	General TB care	Not specified	None
131	Oladimeji 2022	Nigeria	Treatment Completion/Adherence	Pediatric<br>Adult	PLWH
132	Oliwa 2020	Kenya	Diagnosis & Treatment Initiation	Pediatric	None
133	Omar 2024	Somalia	Treatment Completion/Adherence	Adult	None
134	Oonyu 2022	Uganda	TB Preventive Treatment	Pediatric<br>Adult	PLWH
135	Phiri 2021	Malawi	General TB care	Adult	None

136	Phyo 2019	Myanmar	Active Case Finding	Pediatric Adult	Household contacts
137	Pradhan 2010	India	Diagnosis & Treatment Initiation	Adult	None
138	Pradipta 2021	Indonesia	Treatment Completion/Adherence	Adult	Treatment adherence issues/ poor treatment outcomes
139	Pradipta 2022	Indonesia	Diagnosis & Treatment Initiation<br>Treatment Completion/Adherence	Not specified	None
140	Prakoso 2023	Indonesia	Diagnosis & Treatment Initiation	Not specified	Diabetes
141	Probandari 2019	Indonesia	General TB care	Not specified	None
142	Purohit 2015	India	Diagnosis & Treatment Initiation	Not specified	None
143	Reddy 2020	India	TB Preventive Treatment	Pediatric Adult	PLWH
144	Reta 2018	Ethiopia	General TB care	Adult	None
145	Roscoe 2020	Namibia	TB Preventive Treatment	Pediatric Adult	PLWH
146	Roy 2015	India	Treatment Completion/Adherence	Not specified	None
147	Ruru 2018	Indonesia	Treatment Completion/Adherence	Adult	None
148	Rutherford 2013	Indonesia	Treatment Completion/Adherence	Adult	None
149	Salazar-Austin 2021	South Africa	TB Preventive Treatment	Pediatric	Household contacts
150	Sardar 2010	India	Treatment Completion/Adherence	Adult	PLWH
151	Sarwar 2023	Bangladesh	Active Case Finding	Adult	PLWH
152	Serapelwane 2016	South Africa	Treatment Completion/Adherence	Adult	None
153	Shah 2024	India	Diagnosis & Treatment Initiation<br>Treatment Completion/Adherence	Not specified	None
154	Shamanewadi 2020	India	Active Case Finding	Not specified	None
155	Sharma 2015	India	General TB care	Not specified	None
156	Shewade 2018	India	Diagnosis & Treatment Initiation	Not specified	None
157	Shitol 2023	Bangladesh	Diagnosis & Treatment Initiation	Pediatric	None
158	Simbwa 2021	Uganda	General TB care	Not specified	None
159	Singh 2017	India	TB Preventive Treatment	Pediatric	Household contacts

160	Singh 2017	India	General TB care	Not specified	None
161	Singh 2021	India	Diagnosis & Treatment Initiation	Pediatric	Malnutrition
162	Skinner 2016	South Africa	Diagnosis & Treatment Initiation<br>Treatment Completion/Adherence	Not specified	None
163	Skordis-Worrall 2010	South Africa	Diagnosis & Treatment Initiation	Adult	PLWH
164	SouzaLLL 2021	Brazil	Treatment Completion/Adherence	Adult	None
165	Sreevidya 2025	India	TB Preventive Treatment	Pediatric	Household contacts
166	Sultana 2022	Bangladesh	Diagnosis & Treatment Initiation<br>Treatment Completion/Adherence	Pediatric<br>Adult	None
167	Suseendar 2024	India	General TB care	Not specified	None
168	Sylvia 2017	China	Diagnosis & Treatment Initiation	Not specified	None
169	Tadesse 2013	Ethiopia	Diagnosis & Treatment Initiation	Adult	None
170	Teklay 2016	Ethiopia	TB Preventive Treatment	Adult	PLWH
171	Tesfaye 2020	Ethiopia	Active Case Finding	Pediatric<br>Adult	Household contacts
172	Thomas 2020	India	Diagnosis & Treatment Initiation	Adult	None
173	Tupasi 2016	Philippines	Treatment Completion/Adherence	Adult	None
174	Tusho 2025	Ethiopia	Treatment Completion/Adherence	Adult	None
175	vandeWater 2023	South Africa	TB Preventive Treatment	Pediatric<br>Adult	PLWH<br>Household contacts
176	VanGinderdeuren 2019	South Africa	TB Preventive Treatment	Adult	PLWH
177	Vanleeuw 2020	South Africa	General TB care	Adult	None
178	Vanqa 2021	South Africa	Diagnosis & Treatment Initiation	Adult	None
179	Vijay 2010	India	Treatment Completion/Adherence	Adult	None
180	Vijayageetha 2019	India	Active Case Finding	Adult	Pregnancy
181	Wakjira 2023	Ethiopia	General TB care	Adult	None
182	WambiyaEOA 2018	Kenya	TB Preventive Treatment	Not specified	PLWH
183	Watermeyer 2019	South Africa	General TB care	Adult	None

184	Yellappa 2016	India	Treatment Completion/Adherence	Adult	None
185	Yuen 2021	Peru	TB Preventive Treatment	Pediatric<br>Adult	PLWH<br>Household contacts<br>People living with medical comorbidities
186	Zaidi 2024	India	Treatment Completion/Adherence	Adult	None
187	Zhang 2020	China	Treatment Completion/Adherence	Adult	None
188	Zhou 2022	China	Diagnosis & Treatment Initiation	Adult	None
189	Zhou 2024	China	General TB care	Not specified	None

Appendix S5: Descriptive statistics on included studies

Three countries contributed to more than 50% of the included publications: India (n=38; 20%), South Africa (n=38; 20%), and Ethiopia (n=22; 12%).

Among the included studies, the type of FLHCW cadre reported included doctors (n=101; 53%), nurses (n=82; 43%), community health workers (n=38; 20%), pharmacists (n=19; 10%), TB focal point persons (FLHCWs dedicated to providing TB services in a primary care or non-TB specialty setting; n=11; 6%), and other cadres (n=67; 35%). One hundred (53%) papers described barriers specifically related to adults, and 42 (22%) described barriers related to pediatric populations (defined as <15 or <18 years depending on the study).

In comparing the three countries that contributed the most studies to this scoping review, knowledge and skills gaps were commonly reported across India (27/38; 71%), South Africa (22/38; 58%), and Ethiopia (17/22; 77%). Similarly, patient-provider communication challenges were also commonly reported (South Africa: 20/38; 53%, Ethiopia: 9/22; 41%, India: 12/38; 32%). Discrimination and stigma from FLHCW while providing TB services were more frequently reported in South Africa (11/38; 29%) compared to India (3/38; 8%) and Ethiopia (3/22; 14%). Motivation to provide quality care was reported as a barrier in less than a quarter of papers in each of these three countries (South Africa: 6/38; 16%, Ethiopia: 5/22;23%, and India: 5/38; 13%).
